# IGNN: An improved graph neufral network with integrated attention and pre-message-passing for few-shot image classification

**DOI:** 10.1371/journal.pone.0348057

**Published:** 2026-04-28

**Authors:** Jianxiong Chen, Bingwei Fu, Lin Zou

**Affiliations:** 1 Institute of Data Science and Behavioral Science, Civil Aviation Flight University of China, Guanghan, China; 2 Faculty of Science, Civil Aviation Flight University of China, Guanghan, China; Dayananda Sagar College of Engineering, INDIA

## Abstract

Graph Neural Network (GNN) faces limitations in few-shot image classification due to insufficient adaptive feature extraction and limited long-range dependency modeling. To address these challenges, this study proposes an Improved Graph Neural Network (IGNN) integrating two key innovations. Firstly, we design an Attention-Enhanced Feature Extraction module, which combines Efficient Channel Attention (ECA) and self-attention mechanisms, enabling the model to dynamically focus on discriminative intra-image details and inter-image contextual relationships, thereby improving feature representation robustness. Secondly, we introduce a gated recurrent unit (GRU)-based Pre-message-passing mechanism, which establishes cross-sample associations between support and query sets before message propagation, effectively capturing long-range dependencies and mitigating information smoothing. The experimental results of three public datasets demonstrate that our proposed framework outperforms the existing methods and shows significant potential. It offers a pragmatic tool for applications requiring rapid adaptation to limited data, such as remote sensing and medical image analysis.

## 1 Introduction

At present, deep learning models are widely applied in various fields, achieving significant success in supervised learning tasks [1] such as object tracking [2], object recognition [3], sentiment analysis of text [4], image classification [5], image retrieval [6], gesture recognition [7] and engineering application [8]. However, the success of these models fundamentally relies on training with a large amount of labeled data. When data in certain fields is difficult or costly to obtain, and when the total amount of data is large but the labeled data is limited, the advantages of deep learning models are often difficult to demonstrate. To address this issue, the concept of Few-Shot Learning (FSL) [9] has been proposed. FSL is designed to learn certain prior knowledge and then quickly adapt to new tasks with minimal training samples, aiming to achieve effective model training and prediction using only a small number of labeled examples.

Few-shot learning typically employs a meta-learning framework for training, where the model is trained by randomly sampling multiple training tasks from the dataset. Each task consists of a support set and a query set, which are used for training in the inner loop and outer loop, respectively. Through this approach, the model can develop a learning-to-learn capability that enables it to generalize well when facing unseen tasks. Few-shot learning methods can be broadly categorized into three types: metric-based approaches [10,11], memory-based approaches [12–14], and learning-based approaches [15–17]. In recent years, research based on Graph Neural Networks (GNN) has also gained widespread attention in few-shot learning. These methods typically employ GNN for label propagation, using node labels for prediction [18,19], or edge labels for prediction [20,21]. Within this framework, the feature extraction model and classifier are trained simultaneously in both the inner loop and outer loop, and then the output of the feature extraction is used as labels for generating classes. GNN performs feature extraction and classification by passing and aggregating information between nodes in the graph through a message-passing mechanism. The message-passing mechanism of GNN has some limitations. GNN relies on the features of local neighboring nodes for information aggregation, which means it primarily focuses on the transmission of local information, and thus cannot effectively capture the dependencies between distant nodes in the graph. For graph structures that include long-range dependencies, GNN often struggles to provide effective solutions [22]. As the number of layers for information propagation increases in the graph, the representations of nodes may tend to converge, leading to over-smoothing of node features. This can reduce the distinguishability between nodes, thereby affecting the accuracy of graph classification or node classification. Especially in deep networks, message passing can cause node features to gradually become similar, resulting in “information mixing,” which ultimately leads to performance degradation [23].

**Fig 1:**
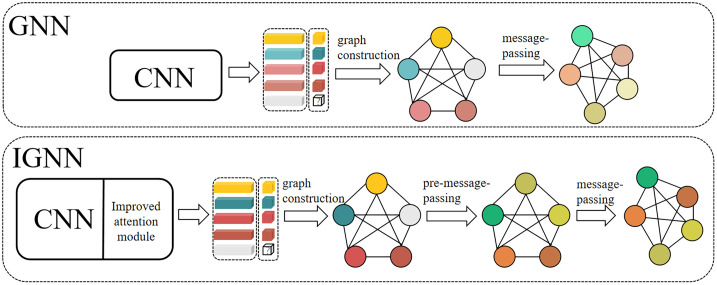
Comparison between traditional GNN and the IGNN proposed in this paper. The IGNN adds an improved attention module and a pre-message-passing mechanism on top of the traditional GNN. The grey circles represent query set sample nodes, while the circles in other colors represent support set sample nodes.

Although existing studies have shown that GNN has certain potential in few-shot learning, the accuracy of current method in tasks such as image classification still lags behind other models. The root of this issue may lie in two aspects: On one hand, the limitations of the message-passing mechanism result in an insufficient ability to capture long-range dependencies; On the other hand, within the framework of meta-learning, there has been ongoing debate over whether rapid learning or feature extraction is more important [24,25]. The importance of rapid learning is self-evident, but good feature extraction is the foundation for rapid learning [26]. We speculate that due to the combined effect of these factors, the performance of GNN in few-shot learning is hindered. Especially in tasks such as image classification and other tasks that rely on global information, the classification performance is difficult to reach an ideal level. To address these shortcomings, as shown in Fig 1, this study proposes an improved graph neural network model named IGNN. Our contributions are as follows:

1. We propose an improved attention module that enables the model to not only focus on internal details of the image but also connect feature information across images during feature extraction. This improvement can effectively enhance the feature representation and information aggregation capabilities of the model, allowing it to better focus on important features and relationships between samples.
2. We introduce a pre-message-passing mechanism to enhance the ability of Graph Neural Networks in capturing complex relationships between nodes, significantly improving the limitations of GNN in information aggregation.
3. We incorporate a pre-message-passing mechanism before the message-passing step in GNN. In the pre-message-passing phase, Gated Recurrent Unit (GRU) is utilized between samples in the support set and query set to address the issue of GNNs overlooking long-range dependencies.

## 2 Related work

### 2.1 Graph neural networks in few-shot learning

Garcia et al. [18] were among the first to propose using GNN for few-shot classification, where each instance is represented as a node in a graph, and the node features are constructed by instance feature and label embedding. The model propagates information through the GNN and finally outputs classification results via a linear classification layer. Liu et al. [19] proposed using label propagation in the transductive setting, leveraging the manifold structure of data by learning the graph structure to propagate labels from support nodes to query nodes. This approach effectively improves the classification performance in few-shot learning. Kim et al. [20] proposed the Edge-Labeling Graph Neural Network (EGNN), which does not directly predict node labels but instead learns edge labels in the graph to infer relationships between nodes, thereby enhancing classification performance in the transductive setting. Luo et al. [27] proposed a Continual Graph Neural Network based on EGNN, which models long-term inter-task correlations and short-term intra-class adjacency, enabling the model to leverage important prior knowledge in new tasks. Yang et al. [21] proposed the Distribution propagation graph network (DPGN). DPGN combines point graphs and distribution graphs to simultaneously learn instance-level and distribution-level relationships, thereby providing finer-grained feature representations and classification decisions.

### 2.2 Feature extraction model

In the related work of few-shot learning, the foundational model for feature extraction plays a crucial role. How to effectively handle contextual semantic information in images is a worthy direction for research. Standard convolution [28] uses a fixed-size convolutional kernel to slide over the feature map, extracting features by performing weighted summation on local regions. Standard convolution can effectively capture local spatial features and has demonstrated strong feature extraction capabilities in tasks such as image recognition and object detection. The weights of the convolutional kernel are updated during training, which enables the model to learn efficient representations for different features. Depthwise separable convolution [29] decomposes standard convolution into depthwise convolution and pointwise convolution, significantly reducing the computational cost. Depthwise convolution performs convolution separately within each channel, while pointwise convolution uses a 1x1 kernel to fuse features across different channels. This approach maintains the feature extraction effectiveness of standard convolution while improving computational efficiency. Residual network (ResNet) [30] builds deep models by introducing skip connections in each convolutional module. This structure allows inputs to be directly propagated to the output, which mitigates the vanishing gradient problem. In the residual modules of ResNet, features are added to the input features after multiple convolutional operations, forming a residual connection. Densely Connected Convolutional Networks (DenseNet) [31] achieve feature reuse by passing the output of each layer to all subsequent layers, forming a dense connection structure. The input of each layer includes not only the output of the previous layer, but also the outputs of all preceding layers. DenseNet can significantly reduce the number of parameters while maintaining high feature transfer efficiency. Deformable convolution [32] adds learnable offsets to standard convolution, allowing the sampling locations of the convolutional kernel to dynamically change based on the input features, thereby adapting to geometric deformations in the image. Compared to traditional fixed convolutional kernels, deformable convolution can more flexibly capture features of irregular shapes.

### 2.3 Attention mechanism

The attention mechanism can play a crucial role in extracting sample features by focusing on internal details of samples as well as information between samples. Squeeze-and-Excitation Networks (SE-Net) [33] introduce a channel attention mechanism, which compresses the global information of each feature channel into a scalar through global average pooling, then uses two fully connected layers to learn the weight for each channel, and finally obtains weight coefficients through the Sigmoid activation function to reweight the features. The SE-Net can effectively distinguish between important and irrelevant features, enabling the model to focus more on discriminative channel information under few-shot conditions. To help the model focus on discriminative spatial regions and feature channels during the feature extraction phase, Woo et al. [34] combined channel attention and spatial attention to propose the Convolutional Block Attention Module (CBAM). The channel attention module first extracts global features through global average pooling and global max pooling, respectively, and then learns the weights for each channel through fully connected layers. The spatial attention module pools the feature maps of each channel. It combines the results of average pooling and max pooling, and then uses a convolutional operation to generate a spatial attention map. However, the dual attention mechanism of CBAM is relatively complex and computationally expensive. Subsequently, efficient channel attention (ECA) [35] is a lightweight channel attention mechanism designed to reduce computational overhead. ECA achieves weight learning across channels using a one-dimensional convolution instead of fully connected layers, thereby adjusting the importance of each channel while avoiding a significant increase in computational cost. Chen et al. [36] combined multi-scale visual Transformer architecture, integrating ECA into the CrossViT model. By leveraging cross-attention, features of different scales are fused together, enhancing interactions between features. This demonstrates that attention mechanism can selectively focus on local details and global information within the input features. Therefore, when extracting features, we need to consider utilizing attention mechanism to effectively integrate local details and global information of the input features, thereby enhancing the comprehensiveness and accuracy of feature representation.

### 2.4 Recurrent neural network

Long Short-Term Memory (LSTM) [37] is a special type of Recurrent Neural Network (RNN) that addresses the issues of gradient vanishing and exploding in the traditional RNN by introducing a gating mechanism. LSTM consists of the input gate, the forget gate, and the output gate. The input gate controls the inflow of new information, the forget gate determines which historical information to discard, and the output gate regulates the output of the current cell state. The structure of LSTM can effectively preserve long-term information, which makes it suitable for learning tasks involving long sequences. Gated Recurrent Unit (GRU) [38] is a simplified version of LSTM, reducing the three gates of LSTM to two: the update gate and the reset gate. The update gate determines how much of the past information to retain, and the reset gate controls how to integrate the new input with the past state. Compared to LSTM, GRU has a simpler structure and higher computational efficiency, making it suitable for resource-constrained scenarios. Bidirectional LSTM [39] adds a backward propagation layer to the standard LSTM, enabling the model to utilize both past and future information simultaneously. This bidirectional propagation mechanism allows the model to reference both preceding and subsequent contexts when processing each time step, enhancing the completeness of information. Attention-LSTM [40] combines LSTM with an attention mechanism, using the attention module to weight the outputs of different time steps, enabling the model to focus on key information within the sequence. This method first extracts features using LSTM, and then weights and sums the outputs through an attention layer to focus on the most discriminative moments. Hierarchical LSTM [41] divides the LSTM into a multi-layered structure, which is designed to capture hierarchical information from the data. Each layer of LSTM units captures features at different hierarchical levels, such as word-level, sentence-level, or paragraph-level semantic information, enabling the model to better process structured data. Self-Attention Enhanced LSTM combines the self-attention mechanism, helping the model to more efficiently focus on the most relevant contextual information at each time step. By adding a self-attention layer after the LSTM output, Self-Attention Enhanced LSTM can capture long-range dependencies within sequences, which aids in improved feature extraction for long sequences.

## 3 Model and method

### 3.1 Problem set-up

In few-shot learning, we typically adopt the framework of meta-learning [42]. Within the meta-learning framework, the target task T is divided into multiple training tasks $\overset{i}{\underset{train}{T}}$ and testing tasks $\overset{i}{\underset{test}{T}}$. The meta-learner learns from a set of training tasks $\overset{i}{\underset{train}{T}}$ and is evaluated on a set of testing tasks $\overset{i}{\underset{test}{T}}$. We represent the set of training tasks as $T_{meta-train}=\{\overset{\text{1}}{\underset{train}{T}},\overset{\text{2}}{\underset{train}{T}},.\;.\;.\;,\overset{n}{\underset{train}{T}}\}$ and the set of testing tasks is represented as $T_{meta-test}=\{\overset{n+\text{1}}{\underset{test}{T}},\overset{n+\text{2}}{\underset{test}{T}},.\;.\;.\;,\overset{n+k}{\underset{test}{T}}\}$. Correspondingly, the dataset for a training task $\overset{i}{\underset{train}{T}}$ is $D_{meta-train}=\{D^{\text{1}},D^{\text{2}},.\;.\;.\;,D^{n}\}$, and the dataset for a testing task $\overset{i}{\underset{test}{T}}$ is $D_{meta-test}=\{D^{n+\text{1}},D^{n+\text{2}},.\;.\;.\;,D^{n+k}\}.$ The dataset for each task is $D^{i}=\{(\overset{i}{\underset{support}{D}},\overset{i}{\underset{query}{D}})\}$, where $\overset{i}{\underset{support}{D}}=\{\overset{s}{\underset{\text{1}}{x}},\overset{s}{\underset{\text{2}}{x}},\overset{s}{\underset{\text{3}}{x}}\overset{s}{\underset{n}{,.\;.\;.\;,x}}\}$, $\overset{i}{\underset{query}{D}}=\{\overset{q}{\underset{\text{1}}{x}},\overset{q}{\underset{\text{2}}{x}}\overset{q}{\underset{\text{3}}{,x}}\overset{q}{\underset{m}{,.\;.\;.\;,x}}\}\;$($\overset{i}{\underset{support}{D}}$ is referred to as the support set, and $\overset{i}{\underset{query}{D}}$ is referred to as the query set).

Meta-learning algorithms are trained in an episodic manner. During training, the N-way K-shot paradigm of meta-learning is followed, where the few-shot classification task is defined as a standard N-way K-shot task. Here, N represents the number of classes, and each class contains K samples. Typically, K is a small value (e.g., 1, 5, or 10), and the total number of samples is N*K. During training on each training task $\overset{i}{\underset{train}{T}}$, the corresponding dataset $D_{meta-train}$ generates a support set $\overset{i}{\underset{support}{D}}$ and a query set $\overset{i}{\underset{query}{D}}.$ The algorithm first learns on the support set $\overset{i}{\underset{support}{D}}$ and then makes predictions on $\overset{i}{\underset{query}{D}}.$ The model parameters are updated based on the prediction results for each training task. After training on multiple tasks, the meta-learning algorithm learns how to effectively learn from small-sample datasets. This stage is referred to as the meta-training phase. Subsequently, the model uses the support set$\;\overset{i}{\underset{support}{D}}$ of the dataset $D_{meta-test}$ to construct a classifier, focusing on rapidly learning task-specific parameters during this phase. The performance of the classifier is then evaluated using $\overset{i}{\underset{query}{D}}$, a process referred to as the meta-testing phase. The generalization ability of the meta-learner’s parameter θ is tested on $D_{meta-test}$ and optimized on $D_{meta-train}$, which can be represented by formulas (1) and (2):


$$\begin{array}{c} \overset{q}{\underset{m}{y}}=f\left(\overset{i}{\underset{support}{D}},\overset{q}{\underset{m}{x}};\theta \right),\;\left(\overset{q}{\underset{m}{x}},\overset{q}{\underset{m}{y}}\right)\in \overset{i}{\underset{query}{D}} \end{array}$$ (1)

where $D^{i}=\left\{\left(\overset{i}{\underset{support}{D}},\overset{i}{\underset{query}{D}}\right)\right\},\;D^{i}\in D_{meta-test}$, $\overset{q}{\underset{m}{x}}$ represents samples from the query set, and $\overset{q}{\underset{m}{y}}\;$ represents the true labels corresponding to the samples in the query set.


$$\theta =\text{arg}\underset{\theta }{\text{min}}\;\sum_{D^{i}\in D_{meta-train}}\;\sum_{\left(\overset{q}{\underset{m}{x}},\overset{q}{\underset{m}{y}}\right)\in \overset{i}{\underset{query}{D}}}\;L\left(f\left(\overset{i}{\underset{support}{D}},\overset{q}{\underset{m}{x}};\theta \right),\overset{q}{\underset{m}{y}}\right)$$ (2)

That is, for the parameter θ learned by the meta-learner under a given task, its performance on the test data of $\overset{i}{\underset{query}{D}}$ is optimal when the training data of $\overset{i}{\underset{support}{D}}$ is known.

### 3.2 GNN

Before introducing our model, let’s first review previous approaches to few-shot learning based on traditional GNN, highlighting the differences between our framework and conventional models. Most current approaches utilize GNN as label propagation module, with [18] serving as a representative example. The fully connected graph in this model contains M nodes, where each node represents the feature values of an instance from either the support set or the query set. In the transductive setting, each fully connected graph contains only one instance from the query set. Therefore, for N-way K-shot few-shot learning, the number of nodes in our fully connected graph is M=N*K+1. In the inductive setting, all nodes in the fully connected graph are constructed from instances in the query set. If a graph G has L layers, with M nodes in its l-th layer denoted as $Z^{l}\in R^{M\times (d_{f}+d_{l})}$, where $d_{f}\;$represents the dimension of instance features obtained from the feature extraction network, and $d_{l}$ is the dimension of the label embedding. In other words, each node is composed of the concatenation of the instance’s features and the one-hot encoding of its corresponding label. Then, the initial node $\overset{\left(\text{0}\right)}{\underset{i}{Z}}$ (representing an instance $x_{i}$ with label i) and the node $Z_{l+\text{1}}$ in the l+1-th layer can be expressed by formulas (3) and (4), respectively:


$$\overset{\left(\text{0}\right)}{\underset{i}{Z}}=\left(\delta \left(x_{i}\right),h(i)\right)$$ (3)

$$Z_{l+\text{1}}=G_{l}\left(Z_{l}\right)=\sigma \left(A_{l}\delta_{l}\left(Z_{l}\right)\right)$$ (4)

where δ is the convolutional neural network, h(i) is the one-hot encoding of the label, and σ is the activation function (the Leaky ReLU function is chosen here). During message-passing, $\delta_{l}$ is a linear transformation function, and $A_{l}\in R^{M\times M}$ is the adjacency operator of the l-th layer (a matrix representing the connections between nodes), with its calculation formula shown in (5):


$$\overset{ij}{\underset{l}{A}}=\psi \left(\delta_{l}\left(Z_{i}\right),\delta_{l}\left(Z_{j}\right)\right)$$ (5)

Here, ψ is a neural network that first computes the absolute difference between two vector nodes, followed by applying a multilayer perceptron (MLP) on the absolute difference, as shown in formula (6):


$$\psi \left(\delta_{l}\left(Z_{i}\right),\delta_{l}\left(Z_{j}\right)\right)=\text{MLP}\;\left(abs\left(\delta_{l}\left(Z_{i}\right)-\delta_{l}\left(Z_{i}\right)\right)\right)$$ (6)

The prediction method for the query node $\overset{q}{\underset{l}{Z}}$ is as shown in formula (7):


$$p_{i}=\overset{q}{\underset{l}{Z}}w$$ (7)

where $w\in R^{(d_{f}+d_{l})\times N}$ represents the parameters in the linear classification layer.

From the principles of GNN, it can be seen that their objective is to use the features and labels of the support set as inputs for nodes in the graph and perform information propagation within the graph, enabling the nodes of the query sample to leverage this information for label prediction. In the graph neural networks designed in [20,21,27], the features and labels of the support set are used as edges in the graph, while the functionality of the label classifier remains unchanged.

### 3.3 IGNN

Previous methods relied solely on CNN for extracting features from the support or query sets, and did not pay sufficient attention to the details of sample features. During message-passing in the graph, only MLP was used, without establishing extensive connections between the samples in the support set and query set. Therefore, the IGNN we propose not only focuses on the internal detailed features of samples when extracting features from the support or query sets but also considers the connections between internal features within the support/query sets and between the support and query sets. In designing IGNN, we introduce an attention module and pre-message-passing mechanism to enhance feature extraction and graph structure modeling for few-shot learning. The attention module guides the model to focus on discriminative regions in support samples while suppressing background noise interference, thereby improving feature utilization efficiency under low-shot conditions. Meanwhile, the pre-message-passing mechanism mitigates node sparseness issues inherent in few-shot scenarios by performing lightweight information exchange before formal graph propagation, strengthening the initial correlations between nodes and laying a more stable foundation for subsequent message passing.

**Fig 2:**
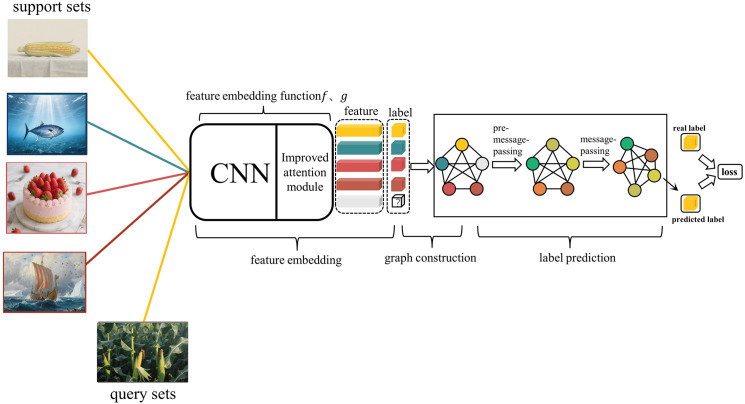
IGNN model workflow diagram. 𝐟 **and** 𝐠 **represent feature embedding functions.** Gray circles represent query set sample nodes, while nodes in other colors represent support set sample nodes. The model first extracts image features from the support set and the query set using the feature embedding functions, and these features are then constructed as nodes in a graph. Next, through the pre-message-passing mechanism, the features of the support set and query set images are associated, and the feature representations of each node are updated. Finally, with the help of the message-passing mechanism, the labels of the query set are predicted, and the predicted labels are compared with the true labels to compute the loss value. The images in this figure are similar to but not identical to the original ones and are therefore for illustrative purposes only.

As shown in Fig 2, the model proposed in this paper consists of three steps: feature embedding, graph construction, and label prediction. In the feature embedding stage, we integrate an improved attention module into the convolutional neural network to further enhance the model’s ability to focus on key channels, thereby extracting features from both the support and query sets. The extracted sample features are used to construct a graph. During image label prediction, we first introduce a pre-message-passing operation to perform preliminary message-passing between the support and query set nodes within the graph structure. This dynamically adjusts the features of the support and query sets, enhancing cross-sample correlations. This design helps the network integrate information in a broader context, which reduces information loss, improves the effectiveness of information transmission and the completeness of feature representation.

#### 3.3.1 Improved attention module.

The ECA module [35] can enhance the feature expression capability of the feature extraction model, enabling better generalization on limited samples. By adaptively assigning weights to different channels, it can more effectively select and emphasize features that are important for classification tasks, which are advantages that can effectively address the challenges of few-shot learning. However, its ability to capture global contextual information is insufficient. To address this limitation, we have improved it. The ECA module generates channel descriptors $H_{c}$ by performing global average pooling on the matrix of the input image feature H, as shown in formula (8):


$$\begin{array}{c} H_{c}=\frac{\text{1}}{N}\sum_{i=\text{1}}^{N}\;H_{i} \end{array}$$ (8)

where N represents the number of nodes, and $H_{i}$ denotes the features of the i-th channel.

To reduce the number of parameters and improve computational efficiency, ECA applies a one-dimensional convolution (${Conv}_{\text{1}}$) to the channel descriptors to generate channel attention weights $\omega_{i}$:


$$\omega_{i}=\sigma \left({Conv}_{\text{1}}\left(H_{c}\right)\right)$$ (9)

Finally, these weights are multiplied element-wise with the input feature matrix to produce the weighted output matrix $H_{e}$, thereby adjusting the input feature matrix and focusing on the details within it, as shown in formula (10):


$$H_{e}=\omega_{i}⨀H$$ (10)

In contrast, the self-attention mechanism generates three distinct matrices—Query (Q), Key (K), and Value (V) by applying linear transformations to the input image features. Where $\omega_{q},\;\omega_{k}$, and $\omega_{v}$ correspond to the learnable weight matrices for these three representations, respectively. This mechanism calculates the similarity S between the query and key to generate the attention weight matrix, and then uses the softmax function to convert it into a probability distribution, thereby highlighting important features. Finally, the attention weight matrix is applied to the value matrix to obtain the attention output $H_{f}$, enabling the contextual information of the support or query set to be integrated into each feature. This allows the model to adaptively adjust feature representations based on the context. Our method combines the advantages of both modules. When extracting features from few-shot samples, it not only focuses on the internal details of the features but also effectively captures the relationships between features.

**Fig 3:**
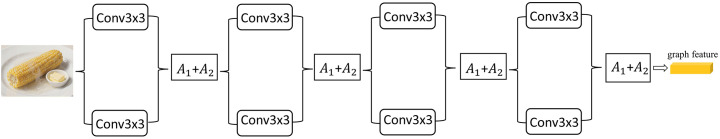
Improved attention module. ${𝐀}_{1}$ **and** ${𝐀}_{2}$ **respectively represent the ECA attention module and the self-attention module.** In the process of extracting image features, four 3 × 3 convolutional layers are used, and modules ${𝐀}_{\text{1}}$ and ${𝐀}_{\text{2}}$ are integrated sequentially after each layer. The images in this figure are similar to but not identical to the original ones and are therefore for illustrative purposes only.


As shown in Fig 3, we use the improved attention module to extract features. First, it enhances the focus on important channels and improves feature representation capabilities to obtain the weighted feature matrix $H_{e}$. Then, it incorporates contextual information into each image feature and results in the matrix $H_{f}$, which integrates both inter-image and intra-image descriptions. This allows the model to simultaneously focus on the details within the features and the relationships between the features. This combination provides stronger feature representation capabilities for few-shot learning, and enhances the model’s performance in various tasks. The computational process is described by the following formulas (11), (12), and (13):

$$Q=H_{e}\omega_{q}\;,\;K=H_{e}\omega_{k},\;V=H_{e}\omega_{v}$$ (11)

$$S=softmax(\frac{QK^{T}}{\sqrt{d_{k}}})$$ (12)

$$\begin{array}{c} H_{f}=SV \end{array}$$ (13)

where $d_{k}$ represents the dimension of the key matrix, used to prevent excessively large values during the softmax function computation.

#### 3.3.2 Pre-message-passing mechanism.

Generally, few-shot learning typically employs two independent embedding functions, $f(\overset{s}{\underset{n}{x}})$ and $g(\overset{q}{\underset{m}{x}})$, applied separately to the support and query sets. These are then connected using a single metric function to link the information from the two embedded images. However, this approach results in features that lack contextual relationships between the support set $\overset{i}{\underset{support}{D}}$ and the query set $\overset{i}{\underset{query}{D}}$. To address this issue, Matching Network [5] first uses standard convolutional neural networks $f(\overset{s}{\underset{n}{x}})$ and $g(\overset{q}{\underset{m}{x}})$ to extract feature values during construction. Then, a bidirectional Long Short-Term Memory network (BiLSTM) $f^{\prime }\left(\overset{s}{\underset{n}{x}}\right)$ is employed to aggregate all features from $f(\overset{s}{\underset{n}{x}})$ into a single vector, enabling the sharing of feature information across all images. A similar method is used to compute $g^{\prime }\left(\overset{q}{\underset{m}{x}}\right)$, but with an attention-based Long Short-Term Memory network (attLSTM) instead. The computation is performed as shown in formulas (14) and (15):


$$f^{\prime }\left(\overset{s}{\underset{n}{x}}\right)=BiLSTM\left([f\;(\overset{s}{\underset{\text{1}}{x}})|\cdot \cdot \cdot |f\;(\overset{s}{\underset{n}{x}})]\right)$$ (14)

$$g\prime \left(\overset{q}{\underset{m}{x}}\right)=attLSTM\left(g\;\left(\overset{q}{\underset{m}{x}}\right),\left\{f^{\prime }\left(\overset{s}{\underset{n}{x}}\right)\right\}\right)$$ (15)

Although both BiLSTM and attLSTM are mechanisms for combining vector sequences into a single vector, attLSTM is order-agnostic, while BiLSTM is order-dependent. Since the elements in the support set are unordered, the dependency in $f^{\prime }\left(\overset{s}{\underset{n}{x}}\right)$ is not well-captured. Furthermore, the treatments of Matching Network treatment for $f^{\prime }$ and $g^{\prime }$ is asymmetric: $f^{\prime }$ depends solely on f , while $g^{\prime }$ depends on both g and$\;f^{\prime }$. Inspired by previous works [5,19,43], the pre-message-passing mechanism we propose not only resolves the issue of $g^{\prime }$ being order-dependent and the asymmetric treatment of the support and query sets but also retains context-aware capabilities. This mechanism is applied to traditional GNN. The core approach involves using GRU for both the support set embedding$\;f^{\prime }$ and the query set embedding $g^{\prime }$. To address the issue that the query set embedding $f^{\prime }$ needs to be defined using the support set embedding $g^{\prime }$, we link these two embeddings through recurrent embedding.

In the initial iteration, we define $f^{\prime }(\overset{s}{\underset{n}{x}})=f(\overset{s}{\underset{n}{x}})$ and $g^{\prime }(\overset{q}{\underset{m}{x}})=g(\overset{q}{\underset{m}{x}})$. Then, using an attention mechanism, we perform L cycles of updates on the embedded $f^{\prime }$ and $g^{\prime }$ (where L is the depth of the graph). This structure allows the embeddings of the dataset to continuously influence the embeddings of the query set during the iterative process. The algorithm is presented in Table 1.

**Table 1:** Pre-message-passing process: Initially, the extracted support set features $f(\overset{s}{\underset{n}{x}})$ and query set features $g(\overset{q}{\underset{m}{x}})$ are used to compute the cosine similarity between the query set and the support set, as well as the similarity among samples within the support set. Then, based on the computed similarities, the features of the support set and query set samples are updated. Subsequently, the updated features are input into GRU for L iterations, resulting in δa and δb. Finally, the embeddings $f^{\prime }$ and $g^{\prime }$ for the support set and query set samples are obtained.

Algorithm
**Define:** $c\;=f(\overset{s}{\underset{n}{x}}),\;\delta a=0,\;\delta b=0,\;f^{\prime }(\overset{s}{\underset{n}{x}})=f(\overset{s}{\underset{n}{x}}),\;g^{\prime }(\overset{q}{\underset{m}{x}})=g(\overset{q}{\underset{m}{x}})$
1: for **i from 1 to** 𝐋 do:
2: $e=𝐜𝐨𝐬\left(g(\overset{q}{\underset{m}{x}})+\delta b,c\right)$, $E\;=𝐜𝐨𝐬(c+\delta a,f(\overset{s}{\underset{n}{x}}))$
3: $a_{j}=\frac{e_{j}}{\sum_{j=1}^{m}\;e_{ij}},\;A_{ij}=E_{ij}\;/\sum_{j=1}^{m}\;E_{ij}$
4: $C=a^{T}c\;,\;c\;=Af(\overset{s}{\underset{n}{x}})$
5: δb=𝐆𝐑𝐔([δb,C]), δa=𝐆𝐑𝐔([δa,c])
6: end for;
7: return $g^{\prime }(\overset{q}{\underset{m}{x}})=g^{\prime }(\overset{q}{\underset{m}{x}})+\delta b,\;f^{\prime }(\overset{s}{\underset{n}{x}})=f(\overset{s}{\underset{n}{x}})+\delta a$

**Fig 4:**
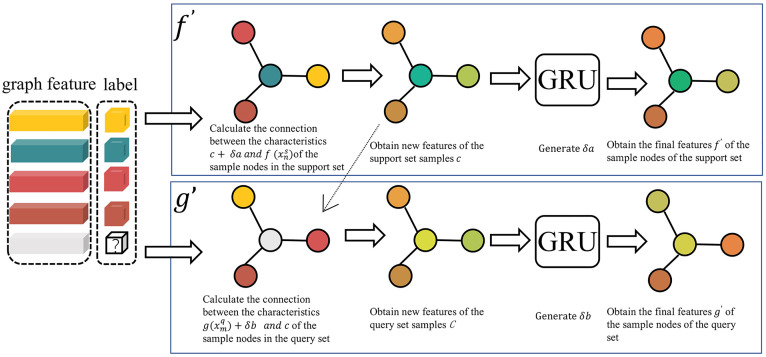
Pre-message-passing mechanism. $f^{\prime }$ represents the pre-message-passing within the support set, while $g^{\prime }$ represents the message-passing between the support set and the query set. Gray circles represent query set sample nodes, and nodes in other colors represent support set sample nodes.

As shown in Table 1 and Fig 4, the pre-message-passing mechanism first extracts initial features from the support and query sets to obtain the support set features $f(\overset{s}{\underset{n}{x}})$ and query set features $g(\overset{q}{\underset{m}{x}}).$ Then, it calculates similarity to assign attention weights. Within the support set, the cosine similarity between support set samples is computed to form the similarity matrix E. Between the support and query sets, the cosine similarity between query set samples and support set samples is calculated to form the similarity matrix e. Attention weights $a_{j}$ and $A_{ij}\;$are generated based on these similarity matrices to adjust the feature representations of the support and query sets.

Perform L iterations using GRU to progressively integrate feature information from the support set and query set. In each iteration, the support set features c are weighted and aggregated based on the attention weights to generate context-aware features C. These features C, along with the current query set features δb, are input into the GRU to update the feature representation of the query set. Simultaneously, the support set features δa are also updated through the GRU to maintain dynamic associations within the support set. After L iterations, the final support set features $f^{\prime }(\overset{s}{\underset{n}{x}})=f(\overset{s}{\underset{n}{x}})+\delta a$ and query set features $g^{\prime }(\overset{q}{\underset{m}{x}})=g^{\prime }(\overset{q}{\underset{m}{x}})+\delta b$ will contain richer cross-sample association information. The pre-message-passing progressively captures global dependencies between the support and query sets through the recurrent mechanism of GRU. The features of the support and query sets influence each other during iterations, avoiding asymmetric treatment in traditional methods and addressing the limitations of local information aggregation in conventional GNN. By leveraging attention mechanisms and similarity calculations, the importance weights of different samples are adaptively assigned, which enhances focus on critical features.

#### 3.3.3 Model training and loss.

During training, we adopt the episodic paradigm. In each training iteration, a random task $\overset{i}{\underset{train}{T}}$ is selected, and a support set $\overset{i}{\underset{support}{D}}$ and a query set $\overset{i}{\underset{test}{D}}$ are sampled from this task. During testing, the accuracy of model is evaluated separately for each test task $\overset{i}{\underset{test}{T}}$. When predicting the label $y_{i}\;$of the image $x_{i}\in T\;\;$ corresponding to node V in the graph, we then compute the cross-entropy loss L at node V [5]:


$$\begin{array}{c} L=-\sum_{i=\text{1}}y_{i}logp\left(\overset{―}{y_{i}}|x_{i}\right) \end{array}$$ (16)

where$\;\overset{―}{y_{i}}$ represents the predicted probability distribution matrix of the label $y_{i}$.

## 4 Experiment

### 4.1 Datasets

When evaluating the performance of our model, we utilized three datasets widely used in the few-shot learning domain: the Omniglot dataset, the MiniImageNet dataset, and the CUB-200–2011 dataset. Below is a detailed introduction to these three datasets:

- Omniglot Dataset: Omniglot is a dataset of handwritten character images containing different alphabets. It includes 1,623 characters from 50 distinct alphabets, with each character class comprising 20 samples written by different individuals.
- MiniImageNet Dataset: MiniImageNet is a streamlined version of the well-known large-scale image dataset ImageNet, consisting of 100 classes and over 60,000 color images in total, with approximately 600 images per class. Compared to Omniglot, MiniImageNet features more complex and diverse images, covering a wide range of objects and scenes.
- CUB-200-2011 Dataset: Fully named Caltech-UCSD Birds-200-2011, this is an image dataset specifically designed for bird species recognition. It contains 11,788 images divided into 200 distinct bird species (200 classes). The number of images per class varies, and each image is accompanied by rich textual descriptions, annotating various attributes such as bird color, shape, etc. CUB-200-2011 is widely used for tasks like object classification, fine-grained recognition, and attribute labeling, especially in few-shot learning for fine-grained classification evaluation.

### 4.2 Experimental settings

#### 4.2.1 Network architecture.

For the feature extraction module, a structure comprising 4 convolutional layers and a fully connected layer is employed to generate 128-dimensional embeddings. The baseline model utilizes a Graph Neural Network. The configurations of the convolutional and fully connected layers are as follows:

First Layer: 3 × 3 convolutional kernel, 64 filters, batch normalization, 2 × 2 max pooling, activation function: Leaky ReLU
Second Layer: 3 × 3 convolutional kernel, 96 filters, batch normalization, 2 × 2 max pooling, activation function: Leaky ReLU
Third Layer: 3 × 3 convolutional kernel, 128 filters, batch normalization, 2 × 2 max pooling, activation function: Leaky ReLU, regularization with Dropout set to 0.5
Fourth Layer: 3 × 3 convolutional kernel, 256 filters, batch normalization, 2 × 2 max pooling, activation function: Leaky ReLU, regularization with Dropout set to 0.5
Fully Connected Layer: 128 neurons, batch normalization

#### 4.2.2 Parameter settings.

In our experiments, we employed the Adam optimizer with β₁ = 0.9 and β₂ = 0.999, which helps balance the first-order and second-order moment estimates for efficient gradient updates. The initial learning rate was set to 1.0 × 10 ⁻ ³, along with a 1000-step warm-up strategy that gradually increased the rate to stabilize the model during early training stages. To prevent overfitting, we applied Dropout during training with a drop rate of 0.5, thereby enhancing the model’s generalization capability. We also implemented L2 regularization (weight decay) with a coefficient of 1.0 × 10 ⁻ ⁴, penalizing the weights to further reduce overfitting risks. The detailed hyperparameter configuration is listed in Table 2.

**Table 2:** Hyperparameter of the model built in our experiments.

Component	Parameter	Value
Optimizer	type	Adam
	β₁	0.9
	β₂	0.999
Learning rate	initial learning rate	1.0 × 10 ⁻ ³
	warm-up steps	1000
Dropout	rate	0.5
L2 regularization	weight decay	1.0 × 10 ⁻ ⁴

#### 4.2.3 Result evaluation.

We conduct experiments with 5-way 1-shot and 5-way 5-shot settings using the three datasets introduced in Section 4.1.

As shown in Table 3, this paper evaluates the proposed model on the Omniglot dataset and compares its performance with other models. The results indicate that the proposed IGNN model achieves the best performance in both 5-way 1-shot and 5-way 5-shot tasks. IGNN can capture detailed sample information in handwritten datasets and is more sensitive to inter-sample relationships, thereby enhancing the performance of traditional graph neural networks.

**Table 3:** Identification accuracy of various models on the Omniglot dataset.

Model	5-way 1-shot	5-way 5-shot
Siamese neural network [10]	97.3%	98.4%
Prototypical network [11]	98.8%	99.7%
Matching network [5]	98.1%	98.9%
Relation network [44]	99.6±0.2%	99.8±0.1%
MAML [15]	98.7±0.4%	99.9±0.1%
MANN [12]	82.8%	94.9%
GNN [18]	99.2%	99.7%
**IGNN**	99.4%±0.05%	99.8%±0.03%

Table 4 presents a performance comparison between IGNN and other models on the MiniImageNet dataset. During training, images from 80 classes are used as the training set, while images from the remaining 20 classes serve as the validation set. All experimental results are averaged after testing. Compared to the Omniglot dataset, MiniImageNet contains richer and more complex information. The proposed IGNN achieves a significant improvement in image classification accuracy over the baseline GNN models. In both 5-way 1-shot and 5-way 5-shot tasks, IGNN outperforms SNAIL by more than 2% and 3%, respectively. These results demonstrate the effectiveness of the proposed feature extraction module and the pre-message-passing mechanism, which address the limitations of traditional graph neural networks and advance technological progress.

**Table 4:** Identification accuracy of various models on the MiniImageNet dataset.

Model	5-way 1-shot	5-way 5-shot
Relation network [44]	50.44±0.82%	65.32±0.7%
Prototypical network [11]	49.42±0.78%	68.2±0.66%
Matching network [5]	46.6%	60%
Semi-supervised PN [45]	50.41±0.31%	64.39±0.24%
MAML [15]	48.7±1.84%	63.11±0.92%
GNN [18]	50.33±0.36%	66.41±0.63%
MANN [12]	57.83±0.69%	71.13±0.50%
AMN [46]	55.30±0.89%	71.80±0.78%
GCR [47]	53.21±0.40%	72.34±0.32%
MACO [48]	41.09%	58.32%
SNAIL [49]	55.71±0.99%	68.88±0.92%
**IGNN**	58.23%±0.65%	72.41%±0.60%

Table 5 shows the performance of the proposed IGNN model on the CUB-200–2011 dataset. Compared to other models, IGNN achieves competitive classification accuracy. Although the CUB-200–2011 dataset is rich in content and has significant overlap between classes, IGNN still performs well. However, because IGNN relies on global graph-level attention mechanisms and propagation schemes, it may overlook critical fine-grained local details. Consequently, when tackling fine-grained recognition tasks like CUB-200–2011, compared to models that focus on part-based modeling (e.g., RCN), it is slightly insufficient. Nevertheless, the improvement of IGNN still highlights the effectiveness in few-shot learning tasks. The results demonstrate that integrating an improved attention module during feature extraction helps extract more representative and robust features from limited data, and the pre-message-passing mechanism positively contributes to graph neural network-based few-shot learning.

**Table 5:** Identification accuracy of various models on the CUB-200-2011 dataset.

Model	5-way 1-shot	5-way 5-shot
MACO [48]	60.76%	74.96%
Muti-scale kronecker product [50]	69.49±0.95%	82.94±0.65%
RCN [51]	74.65±0.86%	88.81±0.57%
MSFN [52]	62.4%	79.14%
LMPNEt [53]	65.59±0.13%	68.19±0.23%
Muti-modal prototypical net [54]	75.01±0.81%	85.30±0.54%
IPN [55]	73.25%	86.81%
**IGNN**	74.37%±0.75%	86.21%±0.70%

**Fig 5:**
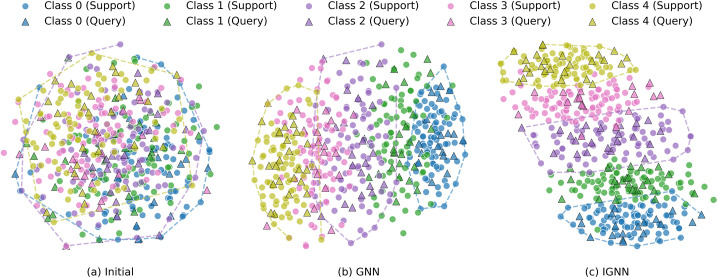
Visualization of node features on the MiniImageNet dataset. The figure represents 5 different classes, with distinct colors corresponding to different classes. Circles represent the support set, and triangles represent the query set. (a) The distribution of different classes before classification; (b) The distribution after classification by GNN; (c) The distribution after classification by IGNN. Compared to GNN, the distribution of nodes with the same color in the support set and query set is more compact in IGNN, indicating better classification performance.

Fig 5 compares the optimization effects of different models on node embeddings via t-SNE visualization using the MiniImageNet dataset. In GNN, samples are not well-clustered, and the boundaries between different classes are relatively blurred. In our proposed IGNN, the improved attention module and pre-message-passing mechanism lead to superior performance in intra-class compactness and inter-class boundary definition, further validating the effectiveness of the proposed modules.

### 4.3 Ablation experiments

To evaluate the effectiveness of the improved attention module and pre-message-passing mechanism proposed in this paper, we conducted ablation experiments on the MiniImageNet dataset using the model, and further investigated the impact of the number of pre-message-passing steps on the final classification accuracy, and provided explanations for the results.

#### 4.3.1 Effectiveness of the improved attention module.

This paper integrates the original attention module and the improved attention module into the baseline model with pre-message-passing, respectively. The classification accuracy of the integrated models on the MiniImageNet dataset is shown in Table 6.

**Table 6:** Comparison of the effects of the ECA module and the improved attention module.

Attention Module	MiniImageNet	
	1-shot	5-shot
ECA	56.41%±0.13%	67.09%±0.34%
Improved Attention Module	58.23%±0.65%	72.41%±0.60%

The experimental results show that the improved attention mechanism significantly improves the model’s classification performance (with a 1.82% increase in accuracy for 1-shot tasks and a 2.32% increase for 5-shot tasks, as detailed in Table 6). This module effectively coordinates the allocation of channel importance and spatial context association in feature representation, enabling adaptive focusing on features in different regions. This combination allows the network to automatically select the most relevant features based on task requirements and facilitates efficient information transmission.

#### 4.3.2 The impact of the number of pre-message-passing steps.

To gain a more comprehensive understanding of IGNN’s performance under different numbers of pre-message-passing steps (L), we modified only the number of pre-message-passing steps in the model and conducted sensitivity analysis experiments on the MiniImageNet dataset, Omniglot dataset, and CUB-200–2011 dataset respectively, and systematically evaluated the impact of varying this parameter from 0 to 8 on model performance.

**Fig 6:**
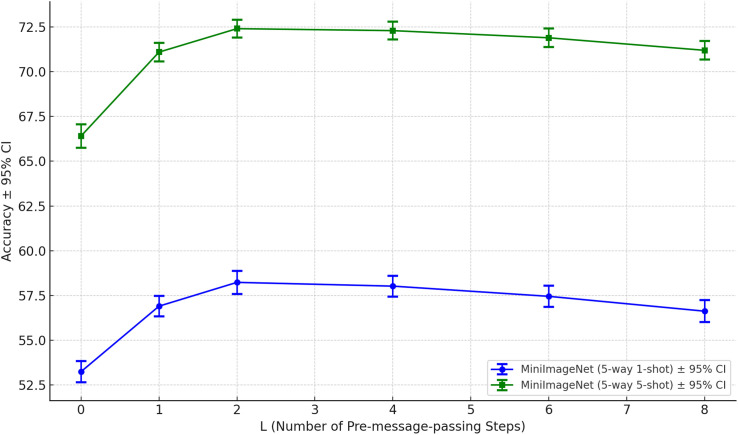
The impact of varying the number of pre-message-passing steps on model performance across the MiniImageNet dataset.

**Fig 7:**
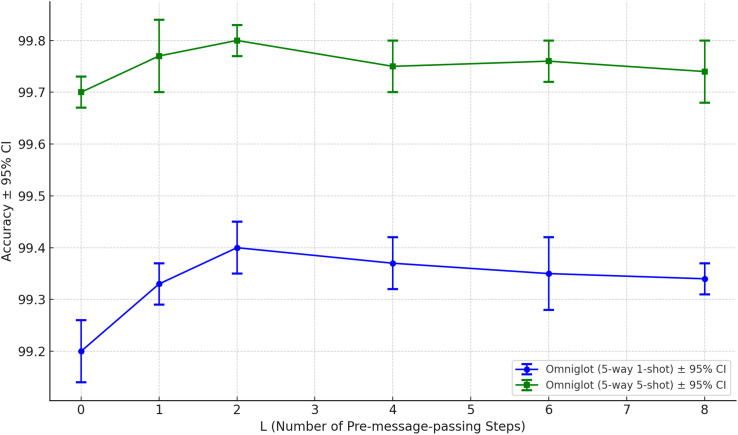
The impact of varying the number of pre-message-passing steps on model performance across the Omniglot dataset.

**Fig 8:**
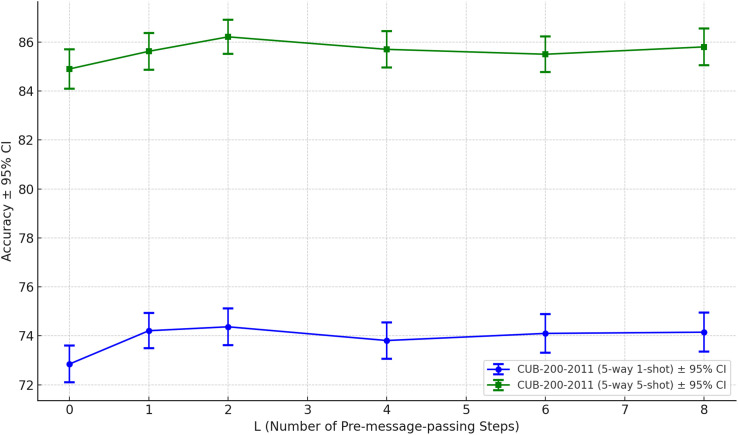
The impact of varying the number of pre-message-passing steps on model performance across the CUB-200-2011 dataset.

The experimental results are presented in Fig 6–8. In the 5-way 1-shot task on the MiniImageNet dataset, IGNN achieved its highest accuracy at L=2. As the number of steps increased further, model performance began to slightly degrade, suggesting that excessive pre-message-passing may cause oversmoothing issues, thereby impairing the capture of fine-grained details. On the Omniglot dataset, IGNN had essentially reached its performance ceiling at L=2, with additional steps yielding negligible gains in accuracy. On the fine-grained recognition task of CUB-200–2011, L=2 also achieved either optimal or most stable performance. When L≥6, a slight decline in accuracy was observed, indicating that excessively deep graph propagation may lead to dilution of local discriminative features, consequently diminishing the model’s ability to distinguish subtle class variations.

We validated both the robustness and performance trends of IGNN across different pre-message passing depths, thereby confirming the rationale for setting L=2 as the default configuration. This configuration not only attained superior performance across multiple datasets but also struck an effective balance between accuracy and computational complexity.

#### 4.3.3 Effectiveness of each module.

To comprehensively analyze the impact of each improvement on the model, we conducted similar controlled ablation experiments on the MiniImageNet dataset. We adopted the same experimental setup as above, using graph neural networks as the baseline model. Three groups of experiments were conducted: (A) Baseline model + Improved attention module, (B) Baseline model + Pre-message-passing mechanism, and (C) Baseline model + Improved attention module + Pre-message-passing mechanism. The experimental results are shown in Table 7.

**Table 7:** The impact of the improved attention module and pre-message-passing mechanism on the baseline model.

Model	MiniImageNet	
	1-shot	5-shot
Baseline Model	50.33±0.36%	66.41±0.63%
A group	53.24%±0.65%	68.12%±0.17%
B group	54.11%±0.60%	69.34%±0.23%
C group	58.23%±0.65%	72.41%±0.60%

The experimental results show that for complex datasets like MiniImageNet, whether adding the improved attention module or integrating the pre-message-passing mechanism, both can significantly improve the classification accuracy of baseline model. Combining both modules leads to a more substantial enhancement in the baseline model’s performance. This further validates that integrating the proposed modules into the baseline model can address the shortcomings of GNN in few-shot image classification tasks, such as insufficient expressiveness in feature extraction and neglect of long-range dependencies during message-passing.

Furthermore, we separately evaluated the inference time and memory usage of the baseline model along with three experimental groups (A, B, and C) on the MiniImageNet dataset. Taking the 5-way-1-shot task as an example, Table 8 displays the measured results per episode—including inference latency, training duration, and peak memory consumption.

**Table 8:** Inference time and memory usage of various models on the MiniImageNet dataset (5-way-1-shot).

Model	Inference latency/ep	Training duration/ep	Peak GPU memory usage	Accuracy±95%CI
Baseline Model	119ms	168ms	3.2G	50.33% ± 0.36%
A group	123ms	174ms	3.3G	53.24% ± 0.65%
B group	125ms	176ms	3.4G	54.11% ± 0.60%
C group	128ms	180ms	3.52G	58.23% ± 0.65%

Compared to the baseline model, IGNN exhibits substantially higher training latency and an increase of approximately 3.2GB in memory usage due to computations in its pre-message-passing and improved attention modules. Despite these additional computational costs, they are deemed reasonable and acceptable when weighed against the improvement in 1-shot accuracy achieved on the MiniImageNet dataset. We contend that IGNN strikes an effective trade-off between precision gains and computational burden, demonstrating particularly notable performance enhancement in the more challenging 1-shot learning scenarios.

## 5 Conclusion

This paper proposes a new few-shot learning model based on graph neural network, named IGNN. Unlike traditional graph neural network, an improved attention module is introduced during the feature extraction process of the graph neural network, which enables the model to focus more on details and critical information during feature extraction. Before the message-passing steps of the graph neural network, we introduce the concept of “pre-message-passing” to capture relationship information between the support set and query set, effectively addressing the issue of traditional graph neural network neglecting long-range dependency information during message-passing. The experimental results demonstrate that our proposed IGNN model achieves significantly superior performance over traditional GNN. While encountering certain limitations in specific fine-grained tasks and challenging environments with intense background noise interference, it still exhibits strong competitiveness in comparison with other models.

## Supporting information

S1 File Code and supplementary data. (DOCX)
