# Supplementary material for: IGNN: An improved graph neufral network with integrated attention and pre-message-passing for few-shot image classification
Source: PLoS One. 2026 Apr 28;21(4):e0348057. doi: 10.1371/journal.pone.0348057 (PMC13123991; doi:10.1371/journal.pone.0348057)
Supplement: S1 File — (DOCX) [file pone.0348057.s001.docx]

**Supporting information**

**Code**

The source code is available at https://github.com/caocaoa/IGNN.git.

**Dataset**

The Omniglot dataset can be accessed from the link:

https://github.com/brendenlake/omniglot.

The MiniImageNet dataset can be accessed [from the link](#OLE_LINK2):

https://image-net.org/update-mar-11-2021.php.

The CUB-200-2011 dataset can be accessed [from the link](#OLE_LINK2):

https://www.vision.caltech.edu/datasets/cub_200_2011/.

**Parameter configuration**

For the parameter configuration of the model on different datasets, including train_N_way, train_N_shots, test_N_way, test_N_shots, batch_size, iterations, and dec_lr were configured via command-line code during experiments and remain adjustable based on task requirements.

| **Configuration** | **MiniImageNet** | | **Omniglot** | | **CUB-200-2011** | |
| --- | --- | --- | --- | --- | --- | --- |
|  | **5-way 1-shot** | **5-way 5-shot** | **5-way 1-shot** | **5-way 5-shot** | **5-way 1-shot** | **5-way 5-shot** |
| train_N_way | 5 | 5 | 5 | 5 | 5 | 5 |
| train_N_shots | 1 | 5 | 1 | 5 | 1 | 5 |
| test_N_way | 5 | 5 | 5 | 5 | 5 | 5 |
| test_N_shots | 1 | 5 | 1 | 5 | 1 | 5 |
| batch_size | 100 | 40 | 100 | 40 | 100 | 40 |
| iterations | 80000 | 90000 | 100000 | 80000 | 110000 | 100000 |
| dec_lr | 15000 | 15000 | 10000 | 10000 | 12000 | 10000 |

**Recognition accuracy across different pre-message-passing steps**

To analyze the effect of pre-message-passing steps (*L*), we systematically tested values ranging from 0 to 8. The resulting performance metrics are summarized in the table below.

| ***L*** | **MiniImageNet** | |
| --- | --- | --- |
|  | **1-shot** | **5-shot** |
| 0 | 53.24%±0.60% | 66.41%±0.65% |
| 1 | 56.90%±0.58% | 71.10%±0.52% |
| 2 (default) | 58.23%±0.65% | 72.41%±0.60% |
| 4 | 58.02%±0.58% | 72.30%±0.50% |
| 6 | 57.45%±0.59% | 71.90%±0.51% |
| 8 | 56.62%±0.61% | 71.20%±0.52% |

| ***L*** | **Omniglot** | |
| --- | --- | --- |
|  | **1-shot** | **5-shot** |
| 0 | 99.20%±0.06% | 99.70%±0.03% |
| 1 | 99.33%±0.05% | 99.77%±0.03% |
| 2 (default) | 99.40%±0.05% | 99.80%±0.03% |
| 4 | 99.37%±0.04% | 99.75%±0.02% |
| 6 | 99.35%±0.05% | 99.72%±0.03% |
| 8 | 99.34%±0.05% | 99.70%±0.03% |

| ***L*** | **CUB-200-2011** | |
| --- | --- | --- |
|  | **1-shot** | **5-shot** |
| 0 | 72.85%±0.75% | 84.90%±0.80% |
| 1 | 74.21%±0.72% | 85.62%±0.75% |
| 2 (default) | 74.37%±0.75% | 86.21%±0.70% |
| 4 | 73.81%±0.74% | 85.70%±0.74% |
| 6 | 73.10%±0.79% | 85.50%±0.73% |
| 8 | 72.45%±0.80% | 84.80%±0.75% |

**Visualization of node features**

**
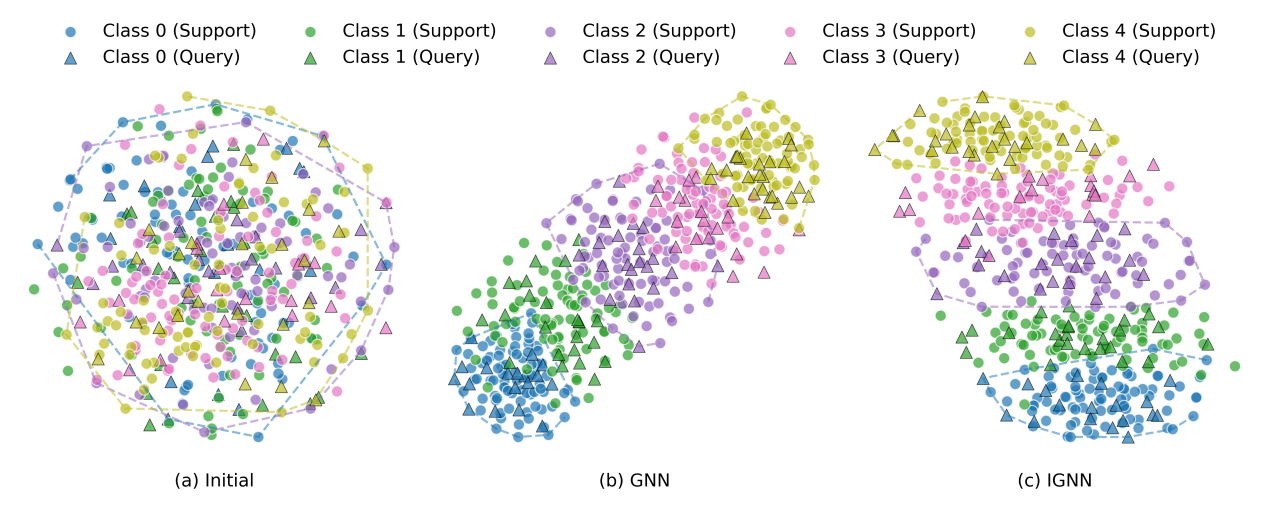
**

Visualization of node features on the Omniglot dataset. The figure represents 5 different classes, with distinct colors corresponding to different classes. Circles represent the support set, and triangles represent the query set. (a) The distribution of different classes before classification; (b) The distribution after classification by GNN; (c) The distribution after classification by IGNN.

**
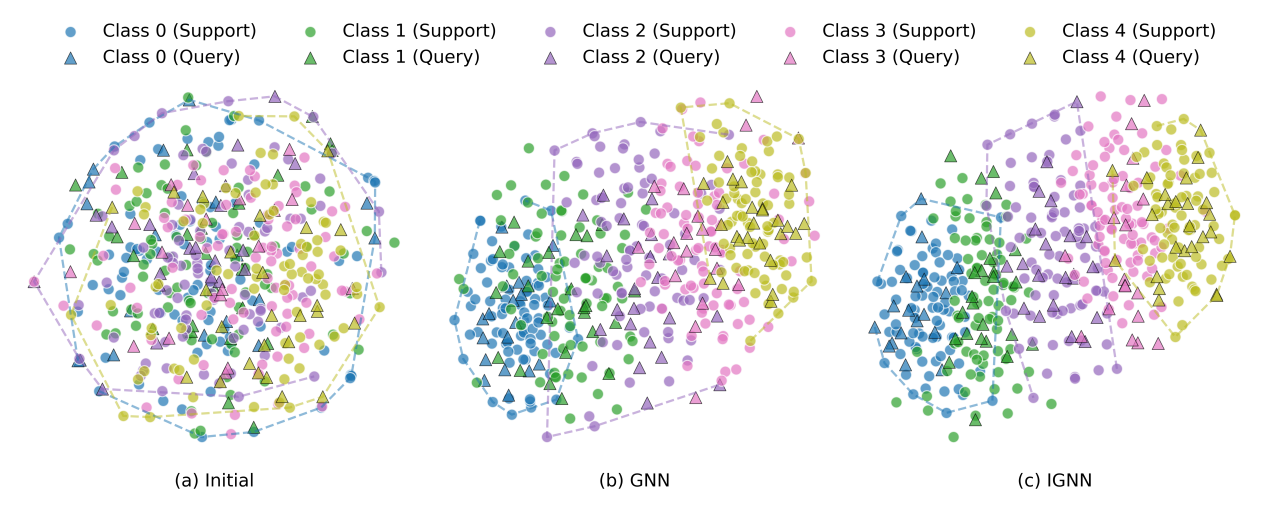
**

Visualization of node features on the CUB-200-2011 dataset. The figure represents 5 different classes, with distinct colors corresponding to different classes. Circles represent the support set, and triangles represent the query set. (a) The distribution of different classes before classification; (b) The distribution after classification by GNN; (c) The distribution after classification by IGNN.
